# Mood and Metabolism: A Bayesian Network Analysis of Depressive Symptoms in Major Depressive Disorder and Metabolic Syndrome

**DOI:** 10.3390/jpm15110563

**Published:** 2025-11-19

**Authors:** Tommaso B. Jannini, Daniele Mollaioli, Susanna Longo, Cherubino Di Lorenzo, Cinzia Niolu, Massimo Federici, Giorgio Di Lorenzo

**Affiliations:** 1 Department of Experimental Medicine, Tor Vergata University of Rome, Via Montpellier 1, 00133 Rome, Italy; 2 Department of Clinical and Experimental Medicine, University of Messina, 98122 Messina, Italy; daniele.mollaioli@unime.it; 3 Department of Systems Medicine, Tor Vergata University of Rome, 00133 Rome, Italy; susanna.longo@uniroma2.it (S.L.); niolu@med.uniroma2.it (C.N.); federicm@uniroma2.it (M.F.); di.lorenzo@med.uniorma2.it (G.D.L.); 4 Department of Medico-Surgical Sciences and Biotechnologies, Sapienza University of Rome, Polo Pontino, 04100 Latina, Italy; cherub@inwind.it; 5 Istituto di Ricovero e Cura a Carattere Scientifico (I.R.C.C.S.)—Santa Lucia, 00179 Rome, Italy

**Keywords:** major depressive disorder, metabolic syndrome, Bayesian network analysis, directed acyclic graph

## Abstract

**Background/Objectives**: Major depressive disorder (MDD) and metabolic syndrome (MetS) are highly prevalent, bidirectionally linked conditions. Individuals with MetS are at increased risk of developing depression, while depression predisposes to metabolic dysfunction. Evidence suggests that comorbid MDD and MetS present a distinct psychopathological profile, with neurovegetative symptoms such as fatigue, sleep disturbances, and appetite dysregulation being more prominent. This study aimed to determine whether depressive symptom structures differ between MDD patients with and without MetS, applying Bayesian network methods to uncover probabilistic dependencies that may inform precision psychiatry. **Methods**: Data were drawn from 1779 adults with ICD-10-diagnosed MDD in the 2013–2020 National Health and Nutrition Examination Survey (NHANES). Using standard metabolic criteria, participants were categorized as MetS (*n* = 315) or non-MetS (*n* = 1464). Depressive symptoms were assessed with the Patient Health Questionnaire (PHQ-9). Directed Acyclic Graphs (DAGs) were estimated via a hill-climbing algorithm with 5000 bootstrap replications to ensure network stability. **Results**: MetS patients displayed a denser and more interconnected symptom network. Fatigue (PHQ4) emerged as a central hub linking sleep, appetite, cognition, and functional impairment. In contrast, non-MetS patients showed a more fragmented network dominated by affective symptoms (low mood, anhedonia) and negative self-cognitions. **Conclusions**: Depressive symptoms propagate differently depending on metabolic status. These results highlight the value of personalized medicine approaches, advocating for treatment strategies that address neurovegetative dysfunctions in MDD with MetS and affective-cognitive symptoms in non-MetS. Aligning interventions with individual symptom architectures and metabolic profiles may enhance therapeutic precision and improve clinical outcomes.

## 1. Introduction

Major depressive disorder (MDD) and metabolic syndrome (MetS) are interconnected conditions with a significant global health impact. MetS is estimated to affect 25% of adults worldwide and is characterized by obesity, dyslipidemia, hypertension, and insulin resistance [1]. Depression impacts over 280 million people globally, with a lifetime prevalence of up to 20% [2].

A bidirectional relationship exists between these two conditions, with individuals with MetS being 1.5 times more likely to develop depression, while depression increases the risk of MetS by 40%. Additionally, genetic predisposition for depression is associated with an increased risk of developing MetS [3].

Emerging theories suggest that depression may not be a homogeneous disorder but rather consists of distinct subtypes shaped by metabolic dysfunction. One influential framework is the concept of “metabolic depression” or “Metabolic Syndrome Type II”, which posits that depressive symptoms, particularly neurovegetative symptoms such as fatigue, anhedonia, and sleep disturbances, may stem directly from underlying metabolic dysregulation [4,5]. These subtypes are characterized by specific biological profiles, such as elevated cytokine levels, increased cortisol, and insulin resistance, which may explain differences in symptom presentation and treatment response.

In this regard, clinical practice highlights how depressive episodes in patients with MetS tend to feature more pronounced neurovegetative and cognitive symptoms compared to emotional or affective symptoms alone. A recent study by Marazziti and colleagues described the “wicked relationship” between depression and MetS, emphasizing that inflammatory markers, altered platelet serotonin, and HPA-axis dysregulation form a vicious cycle of metabolic-psychiatric comorbidity [6]. Similarly, patients with recurrent MDD have been found to show significantly higher rates of MetS compared to first-episode patients, suggesting a cumulative effect of chronic depressive episodes on metabolic health [7].

In line with this evidence, research using general linear model techniques has revealed that certain depressive symptoms, specifically anhedonia and neurovegetative symptoms such as fatigue [8,9], are particularly pronounced in individuals with metabolic syndrome compared to those without. Shared mechanisms, including low-grade chronic inflammation, neuroendocrine dysregulation, and unhealthy habits (such as poor diet, sedentary lifestyle, and smoking), underline this relationship, emphasizing the importance of targeted interventions [10].

The heterogeneity in symptom profiles across individuals with depression suggests a need for more nuanced analytical methods capable of capturing the complex interplay between depressive symptoms and metabolic status. To this end, Bayesian Network Analysis (BNA) offers a flexible, data-driven framework to model conditional dependencies among symptoms. BNA uses probabilistic graphical models, specifically, directed acyclic graphs (DAGs), to infer both symptom connectivity and directional influence, thereby enabling a more refined exploration of comorbidity structures [11,12].

BNA is particularly suited for this research context because it facilitates the identification of central nodes—or “hub” symptoms—that may act as bridges between emotional, cognitive, and somatic dimensions of depression.

The clinical relevance of this approach is profound. By distinguishing the structural organization of symptoms in patients with and without MetS, BNA can inform precision psychiatry—tailoring interventions based on a patient’s specific symptom profile and underlying biological state [13,14].

Given this backdrop, the present study applies Bayesian network techniques to map and compare depressive symptom structures in MDD patients with and without metabolic syndrome, using nationally representative data from the NHANES cohort. We aim to identify divergent pathways and central symptom hubs to guide targeted, mechanism-based interventions for individuals experiencing comorbid psychiatric and metabolic conditions.

## 2. Materials and Methods

### 2.1. Participants, Procedures, and Measures

The present study used publicly available data from the National Health and Nutrition Examination Survey (NHANES), a large-scale, cross-sectional program conducted by the U.S. Centers for Disease Control and Prevention [15]. NHANES integrates comprehensive interviews, physical examinations, and laboratory data to provide a reliable picture of health and disease prevalence across the United States. The strength of this dataset lies in its standardized methodology and the diversity of the sampled population, which enhances the generalizability of findings.

For our analysis, we selected data from four NHANES cycles, spanning the years 2013–2014 through 2017–2020. These cycles were chosen due to the consistent inclusion of key variables relevant to both MDD and MetS. Specifically, the dataset included detailed information on demographic characteristics, physical parameters (such as waist circumference and blood pressure), laboratory values (including fasting glucose, HDL, LDL, and triglycerides), and mental health indicators, most notably the Patient Health Questionnaire (PHQ-9), a widely used screening tool for depressive symptoms [16].

To identify individuals affected by MDD, we focused on participants who were both taking psychotropic medications and had been assigned diagnostic codes consistent with major depressive disorder. More precisely, we included individuals associated with ICD-10 codes F32.9 (Major depressive disorder, single episode, unspecified) and F33.9 (Major depressive disorder, recurrent, unspecified). Additionally, individuals had to complete the PHQ-9 and have available metabolic data.

The diagnosis of metabolic syndrome followed established clinical criteria, consistent with guidelines from the American Heart Association and the International Diabetes Federation [17]. A participant was classified as having MetS if at least three of the following five criteria were met: elevated waist circumference (≥102 cm in men or ≥88 cm in women), high triglyceride levels (≥150 mg/dL), low HDL cholesterol (<40 mg/dL in men or <50 mg/dL in women), elevated blood pressure (systolic ≥ 130 mmHg or diastolic ≥ 80 mmHg), and impaired fasting glucose (≥100 mg/dL). These thresholds align with standard definitions used in epidemiological and clinical studies, ensuring compatibility with previous findings in the field.

By combining both psychiatric and metabolic criteria, we were able to extract a subset of participants who presented with MDD, with or without concurrent metabolic syndrome. Individuals with incomplete data or with conditions potentially confounding the depressive phenotype—such as schizophrenia, bipolar disorder, or psychosis—were excluded from the analysis to ensure sample homogeneity.

### 2.2. Statistical Analysis

We first described the sample using classical univariate statistics (Table 1). For continuous variables such as age, weight, or PHQ-9 scores, we calculated means and standard deviations. For categorical variables like sex or medication status, frequencies and proportions were reported. To compare the MetS and non-MetS groups, we employed Student’s *t*-tests for continuous data and chi-square tests for categorical data. These comparisons allowed us to identify any significant demographic or clinical differences between the two subpopulations and to quantify these differences using effect sizes such as Cohen’s d and odds ratios, where appropriate.

The core of our analytical strategy centered on exploring the internal structure of depressive symptoms using BNA. This method, grounded in probabilistic graphical modeling, is particularly suited to uncovering complex interdependencies between symptoms. Unlike traditional regression models, which often assume that individual symptoms are independent contributors to a latent disorder, BNA treats symptoms as nodes in a network and allows for direct modeling of their conditional relationships.

To construct these networks, we represented the nine PHQ-9 items plus a tenth expressing a functional outcome (“*Difficulty these problems have caused*”) as nodes within a Directed Acyclic Graph (DAG). Edges, or connections, between nodes indicated a statistical dependency between symptoms, with the direction of the arrow representing the potential influence from one symptom to another. The structure of the DAG was determined using a hill-climbing algorithm, a heuristic optimization technique that searches for the most plausible network by minimizing the Bayesian Information Criterion (BIC). The BIC offers a balance between model complexity and goodness-of-fit, thus avoiding overfitting [12].

To ensure the stability and replicability of the inferred network structures, we applied a robust bootstrap procedure consisting of 5000 iterations. This resampling technique generated multiple versions of the data, allowing us to assess how consistently each edge appeared across different samples. Only the most stable connections were retained in the final graphs. Specifically, connections that appeared in at least 85% of bootstrapped networks were considered strong enough to be included, while the direction of the connection had to be consistent in at least 50% of replications to be visualized as a directed edge.

Visualizations of the resulting networks were produced to enhance interpretability. In these figures, the thickness of each edge was proportional to its strength—meaning the statistical contribution of that connection to the overall model. Stronger edges indicated that removing the connection would significantly impair the fit of the model. This approach enabled us to not only detect which symptoms were central within each population’s depressive network but also to infer possible causal sequences among them.

All statistical computations were performed in R (version 4.3.2), an open-source environment widely used for data analysis and visualization. Data preparation and manipulation were conducted using the *tidyverse* package suite (version 2.0) [18]. The Bayesian networks themselves were estimated using the *bnlearn* package (version 4.9.4), a well-established tool for learning and visualizing probabilistic graphical models [11].

By employing these advanced analytical techniques, we aimed to uncover the unique ways in which depressive symptoms organize and interact in individuals with and without metabolic syndrome.

## 3. Results

### 3.1. Participants

From an initial dataset of 60,432 individuals included across four NHANES survey cycles, a total of 1779 participants met the inclusion criteria for this study. All selected individuals had a documented diagnosis of Major Depressive Disorder (MDD), as defined by ICD-10 diagnostic codes (F32.9 or F33.9), and provided complete data on PHQ-9, metabolic biomarkers, and relevant physical parameters.

Within this sample, 315 participants fulfilled the diagnostic criteria for MetS, as outlined by AHA/IDF guidelines. The remaining 1464 participants did not meet the criteria for MetS and thus constituted the comparison group.

Demographic and clinical characteristics of both groups are summarized in Table 1. As expected, individuals in the MetS group had higher body weight, larger waist circumference, elevated blood pressure, higher fasting glucose and triglyceride levels, and lower HDL cholesterol. Importantly, significant differences also emerged in depressive symptomatology between the two groups.

Specifically, individuals with MDD and concurrent MetS reported higher total PHQ-9 scores, reflecting greater overall depressive burden. Upon inspection of individual symptom items, item 3 (“Trouble falling or staying asleep, or sleeping too much”) and item 5 (“Poor appetite or overeating”) were significantly elevated in the MetS group, suggesting a more pronounced expression of neurovegetative symptoms in this population. However, although a statistical difference was reported, all effect size measures were low-modest (not higher than 0.2); fittingly, they have not been considered in the discussion.

### 3.2. Bayesian Network Analysis—Directed Acyclic Graphs (DAGs)

The resulting DAGs revealed different patterns of symptom interconnectivity between the two populations. In the MetS group, the depressive symptom network appeared densely interconnected, with multiple conditional dependencies linking affective, cognitive, and somatic domains. A particularly interesting pathway began with PHQ2 (“*Feeling down, depressed, or hopeless*”), which was connected to PHQ1 (“*Little interest or pleasure in doing things*”) with a moderate arc strength of −42.72. This connection may reflect the classic depressive dyad of low mood and anhedonia. From there, PHQ1 linked directly to PHQ4 (“*Feeling tired or having little energy*”) with an arc strength of −38.37, suggesting that neurovegetative fatigue might follow or co-occur with emotional symptoms.

PHQ4 then emerged as a central hub, radiating outward to a number of other symptoms. It was connected to PHQ3 (sleep disturbances, arc strength = −29.12), PHQ5 (appetite dysregulation, arc strength = −28.47), and PHQ10 (perceived functional impairment, arc strength = −25.76). These connections suggest that fatigue not only reflects core biological disruptions but may also mediate links between mood symptoms and day-to-day functioning. Interestingly, the item expressing appetite changes was further connected to PHQ7 (“*Trouble concentrating*”), with a smaller but still notable arc strength of −21.21, pointing to a potential cascade from somatic to cognitive symptoms in the context of metabolic dysfunction.

In contrast, the network observed in the non-MetS population was sparser and more fragmented, indicating fewer strong conditional dependencies between symptoms. The first was between PHQ1 and PHQ2—anhedonia and depressed mood—with a much stronger arc strength of −266.07, suggesting that these two emotional symptoms are tightly coupled in individuals without metabolic comorbidity. The second strong connection was between PHQ6 (“*Feeling bad about yourself*”) and PHQ9 (“*Thoughts that you would be better off dead*”), with an arc strength of −166.78. This pathway appears to reflect a more cognitive-affective dimension of depression, centered around self-worth and suicidality.

**Figure 1:**
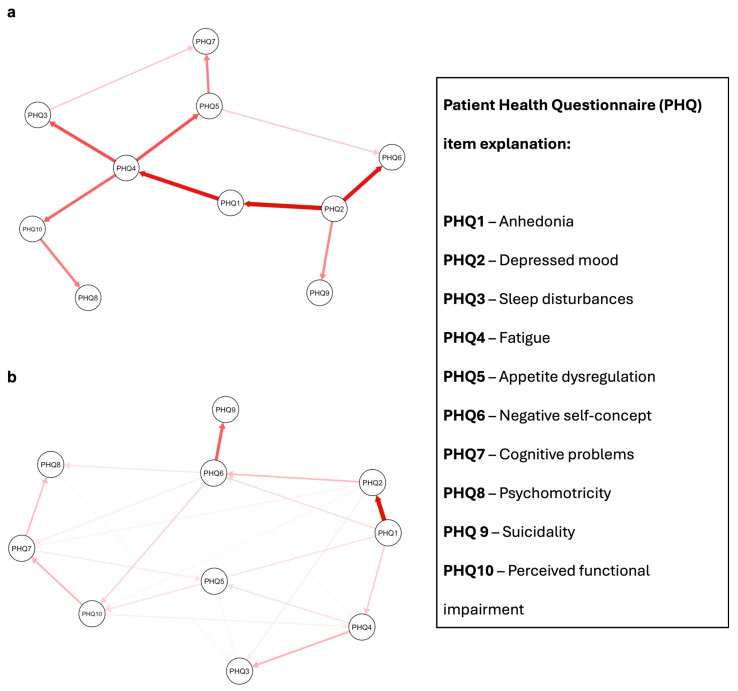
Proposed Directed Acyclic Graphs (DAGs) for depressed patients *with* and *without* metabolic syndrome. Red arrows indicate a causal relationship. Arrow shade refers to arc strength, with more intense colors indicating higher relative importance of each edge in the network. Panel (**a**): depressed patients *with* metabolic syndrome. Panel (**b**): depressed patients *without* metabolic syndrome; PHQ = Patient Health Questionnaire.

The full list of arc strengths, as well as detailed network statistics and bootstrapping metrics, can be found in Table 2 and Table 3. Visualizations of the resulting DAGs are provided in Figure 1, where edge thickness is scaled according to arc strength, offering an intuitive representation of central and peripheral nodes in each network.

## 4. Discussion

The findings of this study provide novel insights into the differential structure of depressive symptomatology in individuals with and without MetS. Leveraging Bayesian network analysis, we demonstrated that individuals with both MDD and MetS exhibit a denser, more interconnected symptom structure, particularly centered around neurovegetative symptoms such as fatigue, sleep disturbance, and appetite dysregulation. In contrast, those with MDD but without MetS display a sparser network, with symptom associations primarily concentrated in affective and cognitive domains.

This structural divergence reflects broader pathophysiological differences that may underlie these two subpopulations. Our findings align with recent Mendelian randomization research by Zhang et al., which showed a causal effect of genetically predicted depression on the risk of developing MetS and several of its components, including waist circumference, hypertension, and dyslipidemia [10]. These findings strengthen the evidence that depression is not merely comorbid with MetS but may actively contribute to its pathogenesis, highlighting the role of depression as a metabolic disruptor.

In individuals with MetS, our analysis revealed that fatigue (PHQ4) occupies a central node in the symptom network, forming strong connections with sleep disturbances (PHQ3), appetite changes (PHQ5), and functional impairment (PHQ10). Fatigue has been recognized as a hallmark feature in the overlap between metabolic and depressive pathology. Mechanistically, it may reflect systemic inflammation, mitochondrial dysfunction, and insulin resistance—core features of both disorders [3]. Studies suggest that chronic low-grade inflammation, particularly elevated interleukin-6 and C-reactive protein levels, may mediate both depressive fatigue and metabolic abnormalities [19].

Further supporting this interpretation is the growing body of literature on “immunometabolic depression”—a subtype of depression marked by prominent neurovegetative symptoms and inflammatory dysregulation [20]. Individuals with this phenotype often display elevated triglycerides, central adiposity, and insulin resistance, all consistent with the clinical characteristics observed in our MetS subgroup.

Interestingly, the connection between appetite disturbances (PHQ5) and cognitive symptoms (PHQ7) in the MetS group adds a novel perspective. This relationship may suggest that metabolic disruptions impact not only somatic domains but also executive function and attentional control. Metabolic dysfunction, especially insulin resistance and elevated inflammatory markers, has been linked to impaired cognitive flexibility and working memory in depressed patients [21,22]. These findings may explain the PHQ5–PHQ7 edge in our DAG, where changes in eating behavior—either as a symptom or consequence of metabolic imbalance—could influence cognitive efficiency.

In contrast, among MDD patients without MetS, symptom pathways appeared more linear and affective in nature. Strong associations were observed between core depressive symptoms such as anhedonia (PHQ1) and depressed mood (PHQ2), as well as between feelings of worthlessness (PHQ6) and suicidal ideation (PHQ9). This pattern reflects what has traditionally been considered the “classic” presentation of depression, where emotional pain and self-referential negative thoughts dominate the clinical picture. These findings also echo cognitive models of depression, which emphasize the role of maladaptive core beliefs and negative attribution styles [23].

The divergent structures in the two networks offer compelling support for the conceptualization of depression not as a monolithic disorder, but rather as a heterogeneous syndrome composed of various overlapping subtypes. The neurovegetative-dominant profile observed in MDD with MetS supports the existence of biologically driven phenotypes that may not respond adequately to standard psychotherapeutic or antidepressant-based interventions. In fact, accumulating evidence indicates that these patients may benefit more from lifestyle modifications, anti-inflammatory agents, or metabolically neutral antidepressants [24,25].

From a clinical perspective, the identification of fatigue (PHQ4) as a central hub symptom in the MetS group holds significant implications as it may foster, similarly to other psychiatric conditions, tailored strategic interventions [26,27,28]. Fatigue’s role as both an outcome and driver of depressive and metabolic symptoms suggests it may serve as a strategic target for intervention. Behavioral interventions such as aerobic exercise have been shown to reduce systemic inflammation and improve energy levels in this population [29]. Similarly, anti-inflammatory pharmacotherapies and dietary interventions such as the Mediterranean diet have demonstrated promise in reducing depressive symptoms in metabolically at-risk individuals [30,31].

Moreover, the increased connectivity in the symptom network of MetS patients suggests that interventions targeting one domain (e.g., fatigue or sleep) may exert downstream effects on a broader constellation of symptoms, potentially yielding a higher treatment payoff. This contrasts with the more fragmented symptom network in non-MetS patients, where symptom-focused treatments may need to be more targeted and psychologically oriented.

The use of Bayesian network analysis in this context offers distinct advantages. Traditional statistical approaches often assume symptom independence or linearity, potentially obscuring the rich interdependencies among symptoms. BNA allows us to model the probabilistic relationships between symptoms, capturing both directionality and strength of influence. In doing so, it provides a more nuanced picture of how depressive symptoms evolve and interact in differing metabolic contexts. This methodology is especially relevant in the age of precision psychiatry, where the goal is not only to diagnose but to tailor interventions based on individual symptom architecture [11,12].

Our findings also offer potential predictive value. For example, in patients with elevated metabolic markers but no current depressive diagnosis, early monitoring of fatigue and sleep disruptions may serve as early warning signs for the development of full-syndrome depression. Conversely, in patients already diagnosed with depression, identifying metabolic risk factors could inform prognosis and treatment selection.

However, although our findings rely on a large sample size, several limitations must be considered. The cross-sectional nature of our dataset precludes definitive conclusions about the causal relationships between depressive symptoms. Although DAGs allow for the inference of potential causal structures, longitudinal data are required to confirm symptom trajectories over time. Furthermore, while our DAG models were statistically robust and bootstrapped over 5000 iterations, external validation in independent datasets is necessary to confirm generalizability. Concerning BNA, DAGs can reveal conditional dependencies among symptoms. However, they cannot determine whether depression + MetS reflects (i) a distinct subtype of MDD characterized by a unique network structure, or (ii) the coexistence of two overlapping conditions, such as somatic symptoms driven by MetS superimposed on depressive symptoms. Second, depressive symptoms were assessed exclusively through the PHQ-9. Although this instrument is widely validated and commonly employed in epidemiological research, its reliance on self-report introduces potential sources of measurement bias. Factors such as social desirability, recall inaccuracies, or cultural differences in symptom expression may have influenced responses. Moreover, self-reported questionnaires may underestimate or overestimate the severity of symptoms in populations with cognitive impairment, alexithymia, or limited health literacy—conditions that are relatively frequent among individuals with metabolic syndrome. Regarding network analysis, we must also acknowledge that a few PHQ9 items are made of a combination of symptoms (e.g., insomnia/hypersomnia or anorexia/hyperphagia, etc.), which warrants extra care when interpreting the findings. This issue might be overcome if such nodes are considered with a generic interpretation rather than a specific symptom (e.g., sleeping problems or appetite changes).

Future works may also consider directly incorporating biomarkers (e.g., CRP, IL-6, HOMA-IR) into the network to map how biological factors interact with symptom pathways. Network studies modelling psychometric measures together with laboratory analytes might help to uncover the multifaceted mechanisms that link clinical presentation and inflammatory dysregulation [32,33]. In addition to network analysis, other statistical approaches may be valuable in investigating the association between depression and MetS. For example, factor analysis could be used to distinguish latent dimensions such as “somatic” and “anhedonia” factors, which could be regressed on MetS-related features. Alternatively, biclustering methods could simultaneously group patients and symptoms to uncover enriched symptom patterns specific to the depression–MetS comorbidity.

Another promising direction is to integrate neuroimaging findings into symptom networks, particularly given that fronto-limbic connectivity has been shown to differ between melancholic and atypical depression subtypes, which may overlap with MetS-related profiles [34].

## 5. Conclusions

In conclusion, this study provides a detailed examination of how depressive symptoms organize differently in individuals with and without MetS, offering a unique contribution to the growing literature on the heterogeneity of depression. By applying BNA, we were able to move beyond traditional categorical or dimensional approaches and to highlight distinct symptom architectures. The denser, neurovegetative-centered network observed in the MetS group underscores the profound influence of metabolic dysfunction on the manifestation and persistence of depressive symptoms. Fatigue, sleep disturbances, and appetite dysregulation emerged as central nodes in this group, suggesting that energy dysregulation and somatic symptoms may serve as key drivers of depression when metabolic abnormalities are present. In contrast, the more affective and fragmented symptom structure identified in the non-MetS group aligns more closely with traditional cognitive-affective models of depression, where low mood, anhedonia, and self-referential negative cognitions dominate the clinical presentation.

At a research level, this study underscores the need for longitudinal and integrative designs. Future work should confirm whether the symptom dependencies identified here remain stable across time and whether targeting central nodes like fatigue can alter downstream symptom trajectories. Incorporating biological markers—such as inflammatory cytokines, cortisol rhythms, or measures of insulin sensitivity—into network models will provide an even more mechanistic understanding of the pathways that link depression and MetS. Furthermore, given the strong bidirectional relationship between these conditions, it will be critical to study whether improvements in metabolic health translate into structural changes in depressive symptom networks, and vice versa [3,22].

## Figures and Tables

**Table 1:** Sample characteristics.

Characteristic	MetS *n* = 315 ^1^	Non-MetS *n* = 1464 ^1^	Effect Size ^2^
Gender			0.91
*Female*	207 (66%)	991 (68%)	
*Male*	108 (34%)	473 (32%)	
Age	54.902 (13.877)	53.389 (16.219)	0.095
Weight (kg)	97.023 (23.011)	87.198 (24.670)	0.402 ***
Waist circumference (cm)	113.984 (15.114)	105.225 (18.059)	0.498 ***
Body Mass Index (kg/m^2^)	34.891 (7.782)	31.878 (8.503)	0.359 ***
Blood Pressure			
*Systolic*	128.671 (20.101)	123.456 (17.429)	0.290 ***
*Diastolic*	73.967 (14.136)	71.271 (11.900)	0.218 ***
Fasting Glucose (mg/dL)	134.025 (53.143)	107.407 (33.795)	0.635 ***
Triglyceride (mg/dL)	192.397 (101.430)	96.338 (50.621)	1.303 ***
Direct HDL-Cholesterol (mg/dL)	43.914 (12.172)	55.397 (16.678)	−0.720 ***
PHQ1 (*Little interest in doing things*)	1.098 (1.062)	1.083 (1.051)	0.014
PHQ2 (*Feeling down, depressed, or hopeless*)	1.219 (1.120)	1.209 (1.062)	0.009
PHQ3 (*Trouble sleeping or sleeping too much*)	1.584 (1.095)	1.374 (1.157)	0.183 **
PHQ4 (*Feeling tired or having little energy*)	1.733 (0.970)	1.633 (1.023)	0.098
PHQ5 (*Poor appetite or overeating*)	1.171 (1.092)	1.036 (1.122)	0.121 *
PHQ6 (*Feeling bad about yourself*)	0.943 (1.196)	0.850 (1.026)	0.088
PHQ7 (*Trouble concentrating on things*)	0.940 (1.111)	0.864 (1.079)	0.069
PHQ8 (*Moving or speaking slowly or too fast*)	0.552 (0.990)	0.479 (0.886)	0.081
PHQ9 (*Thought you would be better off dead*)	0.222 (0.664)	0.241 (0.627)	−0.029
PHQ10 (*Difficulty these problems have caused*)	0.848 (0.935)	0.796 (0.878)	0.057
PHQ Total Score	9.463 (5.857)	8.769 (5.800)	0.119 *

^1^ *n* (%); Mean (SD). ^2^ Cohen’s d is used for continuous variables; Odds Ratio is used for categorical variables. * *p* < 0.05; ** *p* < 0.01; *** *p* < 0.001; MetS = Metabolic Syndrome; PHQ = Patient Health Questionnaire.

**Table 2:** Arc Strength in the Depressed Population *with* Metabolic Syndrome.

From	To	Strength
*PHQ1*	*PHQ4*	−38.3712
*PHQ2*	*PHQ1*	−42.7230
*PHQ2*	*PHQ6*	−38.7644
*PHQ2*	*PHQ9*	−20.4625
*PHQ3*	*PHQ7*	−9.5678
*PHQ4*	*PHQ3*	−29.1284
*PHQ4*	*PHQ5*	−28.4721
*PHQ4*	*PHQ10*	−25.7662
*PHQ5*	*PHQ6*	−9.1726
*PHQ5*	*PHQ7*	−21.2129
*PHQ10*	*PHQ8*	−22.1705

Label: PHQ—Patient Health Questionnaire.

**Table 3:** Arc Strength in the Depressed Population *without* Metabolic Syndrome.

From	To	Strength
*PHQ1*	*PHQ2*	−266.075336
*PHQ1*	*PHQ4*	−58.303795
*PHQ1*	*PHQ6*	−41.475119
*PHQ1*	*PHQ10*	−37.837109
*PHQ2*	*PHQ3*	−23.761115
*PHQ2*	*PHQ6*	−73.165437
*PHQ2*	*PHQ7*	−17.234737
*PHQ2*	*PHQ10*	−12.176333
*PHQ3*	*PHQ5*	−14.913171
*PHQ4*	*PHQ3*	−82.394517
*PHQ4*	*PHQ5*	−38.399162
*PHQ6*	*PHQ4*	−9.286942
*PHQ6*	*PHQ7*	−24.434691
*PHQ6*	*PHQ8*	−33.507499
*PHQ6*	*PHQ9*	−166.782370
*PHQ6*	*PHQ10*	−50.954734
*PHQ7*	*PHQ5*	−28.141069
*PHQ7*	*PHQ8*	−68.776081
*PHQ8*	*PHQ3*	−11.217882
*PHQ10*	*PHQ4*	−22.107131
*PHQ10*	*PHQ7*	−80.191290

Label: PHQ—Patient Health Questionnaire.

## Data Availability

The original data presented in the study are openly available in National Center for Health and Statistics of the Centers for Disease Control and Prevention at https://wwwn.cdc.gov/nchs/nhanes/continuousnhanes/default.aspx (accessed on 11 October 2024).
